# Addiction-Like Mobile Phone Behavior – Validation and Association With Problem Gambling

**DOI:** 10.3389/fpsyg.2018.00655

**Published:** 2018-05-04

**Authors:** Andreas Fransson, Mariano Chóliz, Anders Håkansson

**Affiliations:** ^1^Department of Clinical Sciences, Faculty of Medicine, Lund University, Malmö, Sweden; ^2^Psychology of Addictions, Psychology School, University of Valencia, Valencia, Spain; ^3^Department of Clinical Sciences, Faculty of Medicine, Lund University, Lund, Sweden

**Keywords:** mobile phone, dependence, addiction, behavior, gambling, substance use

## Abstract

Mobile phone use and its potential addiction has become a point of interest within the research community. The aim of the study was to translate and validate the Test of Mobile Dependence (TMD), and to investigate if there are any associations between mobile phone use and problem gambling. This was a cross-sectional study on a Swedish general population. A questionnaire consisting of a translated version of the TMD, three problem gambling questions (NODS-CLiP) together with two questions concerning previous addiction treatment was published online. Exploratory factor analysis based on polychoric correlations was performed on the TMD. Independent samples *T*-tests, Mann-Whitney test, logistic regression analyses and ANOVA were performed to examine mean differences between subjects based on TMD test score, gambling and previous addiction treatment. A total of 1,515 people (38.3% men) answered the questionnaire. The TMD showed acceptable internal consistency (Cronbach's alpha: 0.905), and significant correlation with subjective dependence on one's mobile phone. Women scored higher on the TMD and 15-18 year olds had the highest mean test score. The TMD test score was significantly associated with problem gambling, but only when controlling for age and sex. Various separated items related to mobile phone use were associated with problem gambling. The TMD had acceptable internal consistency and correlates with subjective dependence, while future confirmatory factor analysis is recommended. An association between mobile phone use and problem gambling may be possible, but requires further research.

## Introduction

In recent years, behavioral addictions as clinical diagnoses have been given more interest and validity, marked by gambling disorder (GD) being accepted in the latest edition of DSM-5 in the chapter of “substance-related and addictive disorders”, reflecting its resemblance to substance use disorder (American Psychiatric Association, 2011). The prevalence of GD has been estimated to be between 0.4 and 1.1%, somewhat depending on survey methods and the population studied (Götestam and Johansson, 2003; Petry et al., 2005; Toce-Gerstein et al., 2009; McBride et al., 2010). Petry and co-workers showed that the lifetime prevalence of GD was 0.42% in a large American study (Petry et al., 2005). High rates of lifetime comorbidities between GD, alcohol and other substance use disorders, as well as other psychiatric disorders have been found in several studies, highlighting possible implications for treatment and outcome in these individuals (Petry et al., 2005; Dowling et al., 2015; Jiménez-Murcia et al., 2016). Moreover, many studies have shown that there exist sex differences in GD (Ladd and Petry, 2002; Svensson and Romild, 2014). In a large Swedish representative sample study it was revealed that male sex was related to a higher risk of developing GD, together with being younger than 25 years old (Abbott et al., 2014). Although, while more commonly seen in young men, GD tends to have a later onset in women, and women also generally gamble for a shorter period of time before seeking professional treatment in relation to their gambling (Ladd and Petry, 2002).

Along with GD being accepted in DSM-5, Internet Gaming Disorder (IGD) was mentioned as a condition warranting more clinical research and experience before it might be considered for inclusion in the main book as a formal disorder (American Psychiatric Association, 2013; Petry et al., 2015). Thus, this gives support to the idea that further non-substance-related conditions could be considered as potential addictive disorders, and that associations between them might be present.

The increased attention being directed toward behavioral addictions has during recent years also brought interest to the use of mobile phones. According to a survey made in 2017, 98% of the Swedish population owned a mobile phone. The majority of these were smartphones, facilitating Internet access, the use of applications of social networks, gaming and gambling (Davidsson and Thoresson, 2017).

The fact that almost everyone today owns a mobile phone, and that Internet access is possible virtually everywhere, has sparked an interest within the research field in how this might affect patterns of use. Some studies have examined potential benefits in using mobile phone services to support healthy behaviors, and in providing health interventions to different patient groups (Head et al., 2013; Pfammatter et al., 2016; Posadzki et al., 2016; Adler et al., 2017; Armanasco et al., 2017). However, several other studies have focused on adverse aspects and possible consequences of increased mobile phone use. Associations have been found between intense mobile phone use and mental distress, such as depression, anxiety, stress and sleep disturbances (Yen et al., 2009; Thomée et al., 2011; Demirci et al., 2015; Exelmans and Van den Bulck, 2016; Roser et al., 2016; Tamura et al., 2017; Lopez-Fernandez et al., 2018). A Spanish study found that alcohol consumption may predict problematic mobile phone use, which might suggest a possible association between excessive mobile phone use and other addictive disorders (De-Sola et al., 2017b).

The World Health Organization has now raised attention toward potential health implications of excessive mobile phone use, and some researchers have even suggested a possible new form of behavioral addiction (Chóliz, 2010; Billieux et al., 2015a; Haug et al., 2015; Montag et al., 2015; Nikhita et al., 2015; World Health Organization, 2015; Long et al., 2016; Sapacz et al., 2016). Several questionnaires have been developed and tested in trying to measure this concept, generally named “mobile phone dependence” or “mobile phone addiction” within the research field of potential non-substance addictions. These questionnaires are normally based on criteria in the DSM-IV for substance dependence disorder or modified from scales trying to measure problematic internet use (Toda et al., 2004; Bianchi and Phillips, 2005; Billieux et al., 2008; Yen et al., 2009; Chóliz, 2012; Kwon et al., 2013; Kim et al., 2014; Lopez-Fernandez, 2017).

One of the questionnaires developed is the Test of Mobile Phone Dependence (TMD), which has been tested and validated on Spanish and Persian adolescents (Chóliz, 2012; Mohammadi et al., 2015).

However, the concept of mobile phone dependence has not been without controversy within the research field. Some researchers have questioned the presence of a psychopathological concept such as mobile phone dependence, as no established theoretical framework or defined symptomatology exist (Billieux et al., 2015a). Moreover, criticism has been directed at the approach of translating the diagnostic criteria of substance-related and addictive disorders and applying them to a different kind of potential addiction (Billieux et al., 2015b).

To the best of the authors' knowledge, no research has evaluated whether a potential mobile phone dependence—or an addiction-like mobile phone behavior—may be associated with problem gambling, i.e., with the only addictive behavioral patterns so far established as a diagnosis. Since addictive disorders have shown to be associated with one another, there is reason to investigate whether such an association also exists between addiction-like mobile phone behavior and GD. Also, there is need to test potential evaluation instruments for mobile phone dependence in different settings, and to further examine their potential validity and usefulness, not only in the youngest individuals, but in the general population. Therefore, in the present study, the primary aim was to translate and validate an instrument for mobile phone dependence in the Swedish general population, through a web survey distributed through social media and on the Internet, and a secondary aim of the study was to investigate whether there is an association between mobile phone use, problem gambling and an experience of seeking treatment for a gambling problem.

## Materials and methods

### Questionnaire development

#### Descriptive data

The first part of the questionnaire was based on descriptive data collection. Age category and sex of responding subjects was collected, together with basic usage patterns of mobile phone use, such as total calls per day, total text messages per day, and subjective dependence (See Table 1, questions 1–8).

Table 1The questionnaire (Translated from the Spanish version of the TMD, for purpose of the reader).

**Table:** 

*1. On an average day…*				
*a. How many calls do you make with your mobile phone?*				
**0–5**	**6–10**	**11–15**	**16–20**	**More than 20**
				
*b. How many text messages do you send with your mobile phone?*				
**0–5**	**6–10**	**11–15**	**16–20**	**More than 20**
				
*2. Do you usually turn off your mobile phone at night?*				
**Yes**	**No**			
*3. How often do you use your mobile phone communicate with people while in bed?*				
**Never**	**Rarely**	**Sometimes**	**Often**	**Almost every day/night**
				
*4. How often do you use your mobile phone to play free online games, bet on sports or other gambling?*				
**Never**	**Rarely**	**Sometimes**	**Often**	**Almost every day/night**
				
*5. How many people do you communicate with through social media, chat functions, WhatsApp, Skype or other on an average day?*				
**0**	**1–10**	**11–50**	**51–100**	**More than 100**
				
*6. How many hours per day do you usually use social media, chat functions, WhatsApp, Skype or other?*				
**Less than 1 h**	**1–2 h**	**2–3 h**	**3–4 h**	**More than 4 h**
				
*7. How long does it usually take for you to answer through social media in the following situations?*				
	**I answer immediately**	**I wait a little, but try to answer as fast as possible**	**I wait as long as I need until I answer**	**I never answer in this situation**
While relaxing at home				
While at work or in school				
While with friends				
While in bed				
While eating lunch

**Table:** 

*8. On a scale from 0 to 100, to what extent do you think you are mobile phone dependent?*										
**0%**	**10%**	**20%**	**30%**	**40%**	**50%**	**60%**	**70%**	**80%**	**90%**	**100%**

**Table:** 

*9. Indicate to what degree you agree or disagree with the statements below*.					
	**Never**	**Rarely**	**Sometimes**	**Often**	**Frequently**
**Item 1**. Someone criticizes or warns me about using the mobile phone too much					
**Item 2**. I set a limit to my mobile phone use but do not manage to stick to it					
**Item 3**. I argue with my relatives about the cost of my mobile phone use					
**Item 4**. I spend more time than I would like to using my mobile phone					
**Item 5**. I feel that I use my mobile in an excessive manner					
**Item 6**. I go to sleep later or sleep less because of my mobile phone use					
**Item 7**. I spend more money than initially expected on my mobile phone use					
**Item 8**. I use my mobile phone because I am bored					
**Item 9**. I use my mobile phone in situations where it is inappropriate (although not dangerous) to do so					
**Item 10**. I have been criticized because of the cost of my mobile phone use					
*10. Indicate how frequently the statements below apply to you*.					
	**Completely disagree**	**Disagree somewhat**	**Neutral**	**Agree somewhat**	**Completely agree**
**Item 11**. When I haven't used my mobile phone for a while, I feel the need to call someone, send an sms or to contact someone through social media					
**Item 12**. Lately I've been using my mobile phone more and more					
**Item 13**. If my mobile phone were broken for an extended period of time and I wasn't able to use it, I'd feel bad					
**Item 14**. I need to use my mobile phone more and more					
**Item 15**. If I don't have my mobile phone, I feel bad					
**Item 16**. When my mobile phone is in my hand, I can't stop using it					
**Item 17**. It is not enough to use my mobile phone in the way I did when I first got it, I am steadily using it more and more					
**Item 18**. The first thing I do when I get up in the morning is to see if someone has called me, sent me an SMS or written to me through social media					
**Item 19**. I spend more money on my mobile phone now than when I first got it					
**Item 20**. I don't think I could stand 1 week without a mobile phone					
**Item 21**. When I feel lonely, I call someone, send an SMS or send a message through social media					
**Item 22**. Right now I feel like making a call, sending an SMS or sending a message through social media					
*11. Gambling (e.g., online, at a Casino, slot machines or sports betting?)*.					
*Have there ever been periods lasting 2 weeks or longer when you spent a lot of time thinking about gambling experiences or planning out future gambling ventures or bets?*					
**Yes**	**No**				
					
*12. Have you ever tried to stop, cut down or control your gambling?*					
**Yes**	**No**				
					
*13. Have you ever lied to family members, friends or others about how much you gamble and how much money you lost on gambling?*					
**Yes**	**No**				
					
*14. Gaming not concerning money (e.g., computer games, video games, mobile phone games). Have there ever been periods lasting 2 weeks or longer when you spent a lot of time thinking about gaming experiences or planning out future gaming?*					
**Yes**	**No**				
					
*15. Have you ever tried to stop, cut down or control your gaming?*					
**Yes**	**No**				
					
*16. Have you ever lied to family members, friends or others about the extent of your gaming?*					
**Yes**	**No**				
					
*17. Have you ever received professional care, treatment or counseling for gambling problems or gambling disorder?*					
**Yes**	**No**				
					
*18. Have you ever received professional care, treatment or counseling for alcohol- or drug-related problems?*					
**Yes**	**No**				
					

*Descriptive data: 1–8. The TMD: 9–10. NODS-CLiP gambling: 11–13. NODS-CLiP gaming: 14–16. Previous addiction treatment: 17–18*.

#### The TMD

First, the TMD, a 22-item questionnaire originally developed and validated in Spain (Chóliz, 2012), was translated directly from Spanish to Swedish through oblique translation technique (See Table 1, questions 9–10). The maximum score of the TMD was 88 points. Each one of the 22 non-weighted questions, based on a 5-point Likert scale, could yield either zero, one, two, three or four points. Items 1–10 are answered on scales ranging from 0 (*never*) to 4 (*frequently*), while items 12–22 are answered on scales ranging from 0 (*completely disagree*) to 4 (*completely agree*). The cut-off test score for probable mobile phone dependence was set to 66, with a cut-off test score of possible mobile phone problem use set to 58 points. The cut-off scores were adapted from the Spanish version of the test.

#### The NODS-CLiP

Furthermore, the NODS-CLiP, a validated three-question screening instrument for adult pathological and problem gambling was translated from English to Swedish through oblique translation technique and included in the final questionnaire (Toce-Gerstein et al., 2009). A subject having answered “yes” to any one of the three NODS-CLiP gambling items was defined as a “problem gambler.” The NODS-CLiP has been described to have excellent sensitivity and acceptable (around 90%) specificity for the identification of problem gambling in the general population (Toce-Gerstein et al., 2009).

#### Addiction treatment

Finally, two non-compulsory dichotomous (yes/no) questions were added to the final questionnaire, to study if the subject had ever received professional treatment for gambling-, alcohol-, or drug-related problems. The two non-compulsory questions differed from the other questions in the questionnaire in that the subject could choose to leave them unanswered.

#### The final questionnaire

The final version of the Swedish questionnaire, consisting of descriptive data, the TMD, the NODS-CLiP and addiction treatment questions, was subsequently published on a webpage that was accessible online (see Table 1). The questionnaire was voluntary and anonymous, of which the subject was informed at the start page. For the purpose of the statistical analysis, a cut-off for the highest subjective dependence score was arbitrarily set at 80%. After finishing the questionnaire, the subject was presented a total score and a short description of what their total score might indicate, and advice about where to seek professional counseling if needed. As part of the questionnaire, questions similar to the NODS-CLiP, but adapted for gaming rather than for gambling, were also included, but not used further in the study.

### Subjects and study procedure

The study sample was a convenience sample, and the questionnaire was open to any subject with access to the Internet. The questionnaire was accessible online between February 18th and April 6th, 2016. The questionnaire was predominantly distributed through Facebook, Twitter, Google, and E-mail.

Facebook—between the 18th and 20th of February, 2016, a link to the questionnaire was published by the authors on their private Facebook profile pages with an invitation to followers to share the link unconditionally.

Google and E-mail—a list of key words was purchased, related to mobile phone use, gambling and gaming. Among the words purchased were “best mobile phone,” “cheapest mobile phone subscription,” “betting,” “casino,” and “lottery.” E-mails were sent out to the principals of high schools and upper secondary schools in the cities of Landskrona, Lund and Malmö, with an appeal of spreading the questionnaire to their students. A convenience sample was used and principals of 65 schools were e-mailed between the 5th and 10th of March 2016.

Twitter—a twitter account was created, spreading the survey link on twitter pages of gambling companies, using hashtags related to gambling.

The data collection was made by PIB, a Swedish company experienced in online surveys. The sample was not nationally representative. Participants choosing to display the link to the survey had to provide informed consent electronically (but without any identifying personal information) in order to enter the questionnaires. No identifying information was obtained from the survey, and geographical location was not asked for, and age was reported only in 5-year categories, such that both direct and indirect identification would be impossible. The present web survey was entirely self-selective, and presented as a self-test for mobile phone dependence. At the end of the survey, the participants received an automated message indicating whether they scored above cut-off on mobile phone dependence, and including a brief advice to seek professional therapeutic assistance if unable to cut down on a potentially problematic pattern of mobile phone use. The study was approved by Lund regional ethics committee, Sweden (file number 2016/53).

### Statistical analysis

#### Data analysis

Statistical analyses were performed using IBM SPSS Statistics version 23.0 and FACTOR version 10.8.01. Parallel Analysis was conducted through Monte Carlo simulation in SPSS. Polychoric correlation matrix was computed in FACTOR. A *p* level lower than 0.05 was considered statistically significant. Mahalanobis distance was computed before data analysis. Independent samples *T*-tests, ANOVA and Mann-Whitney tests were performed to examine the mean differences between subjects, based on test score on mobile phone use, gambling, age and a history of having received professional treatment for gambling or alcohol- or drug-related problems. Logistic regression analyses were performed to examine odds of scoring above cut-off score on the TMD, and for problem gambling, in relation to sex and age and separate mobile phone use items. Effect size, measured as eta squared (η^2^), was calculated from the ANOVA table in SPSS.

#### Factor analysis

The adequacy of the data for factor analysis was investigated with the Kaiser-Meyer-Olkin measure of sampling adequacy (KMO) and Bartlett's test of sphericity (Tabachnick and Fidell, 2007). Parallel analysis (PA) was conducted to decide the numbers of factors to be retained in the subsequent exploratory factor analysis. Factors with greater eigenvalues, when compared to the simulative matrix with iteration number of 1,000, were considered significant and retained (Horn, 1965; Zwick and Velicer, 1986; Velicer and Jackson, 1990; Tabachnick and Fidell, 2007; Izquierdo et al., 2014). PCA with Promax oblique rotation was performed in FACTOR, based on polychoric correlations. Promax oblique rotation was used with a Kappa value of 4, as it was assumed that the factors would correlate with one another. The pattern matrix was analyzed in relation to item factor loadings. Items with factor loading >0.30 were included in the factors (Floyd and Widaman, 1995). Items with factor loading >0.32 on more than one factor was considered a cross-loading (Costello and Osborne, 2005). In the case of cross-loading the item was decided to belong to the factor with the highest loading, whereby structure matrix was also analyzed together with conceptual reasoning of the assumed latent nature of the item (Coulacoglou and Saklofske, 2017).

#### Reliability analysis

Reliability analysis of the TMD was performed through internal consistency computation of Cronbach's alpha coefficient and considered acceptable if it exceeded 0.7 (Tabachnick and Fidell, 2007).

## Results

### The questionnaire

Upon closing the online questionnaire, 1,769 people had entered the start page. Out of these, 28 people left instantly without proceeding to the questionnaire. Another 8 people were under 15 years old, and were denied to fill out the questionnaire. Out of the 1,769 people who entered the start page of the questionnaire, a total of 1,515 people completed it (men *n* = 580, 38.3%, women *n* = 935, 61.7%). Only the results of the 1,515 subjects who completed the questionnaire were statistically analyzed, after Mahalanobis distance was computed for outliers. Six people chose not to answer the question “Have you ever received professional care, treatment or counseling for gambling problems or gambling disorder?” and 12 people chose not to answer the question “Have you ever received professional care, treatment or counseling for alcohol- or drug-related problems?.” The majority of the subjects answered the questionnaire through the Facebook link (57.6%). The largest number of subjects was found in the age group 15–18 years old (30.3%), while the smallest number of subjects was found in the age group 19–24 years old (9.0%), see Table 2. The average subject made 0–5 phone calls a day, sent 6–10 text messages (Table 3), and communicated with 1-10 people through their mobile phones (Table 4). Three percent of the subjects rated their subjective mobile phone dependence at 100%, and a total of 17.2% of the subjects rated it at 80% or higher.

**Table 2:** Age distribution of the subjects.

**Age group**	**n**	**%**
15–18 years	459	30.3
19–24 years	137	9.0
25–29 years	194	12.8
30–39 years	339	22.4
40–49 years	186	12.3
50 years or older	200	13.2
	1,515	100

**Table 3:** Average daily mobile phone use.

**Number of people**	**0–5**	**6–10**	**11–15**	**16–20**	**>20**	**Total**
On an average day, how many phone calls do you make with your mobile phone? (n, %)	1,125 (80.9)	209 (13.8)	51 (3.4)	12 (0.8)	18 (1.2)	1,515 (100)
On an average day, how many SMS do you send? (n, %)	728 (48.1)	347 (22.9)	159 (10.5)	76 (5.0)	205 (13.5)	1,515 (100)

**Table 4:** How many people do you usually communicate with in a day through social media, chat functions, WhatsApp, Skype, or other?

	***n***	**%**
None	90	5.9
1–10 people	1,201	79.3
11–50 people	203	13.4
51–100 people	12	0.8
More than 100 people	9	0.6
	1,515	100

### Factor analysis

The dataset proved suitable for exploratory factor analysis with a KMO of 0.920 and statistical significance for Bartlett's test of sphericity (p < 0.001). Parallel analysis suggested that three factors should be retained in the subsequent PCA based on polychoric correlations (Table 5). Tables 6, 7 show the rotated loading matrix and structure matrix respectively from the PCA with Promax oblique rotation based on polychoric correlations.

**Table 5:** Parallel analysis based on principal components analysis of the TMD.

**Variable**	**Real-data eigenvalues**	**Mean eigenvalues**	**95 percentile of random eigenvalues**	**Cumulative proportion of variance**
1^*^	9.220	1.306	1.360	41.91 %
2^**^	1.943	1.248	1.291	50.74 %
3^***^	1.590	1.206	1.239	57.96 %
4	1.078	1.173	1.198	
5	0.989	1.144	1.166	
6	0.844	1.120	1.139	
7	0.734	1.094	1.115	
8	0.647	1.070	1.089	
9	0.588	1.048	1.066	
10	0.536	1.026	1.044	
11	0.510	1.004	1.023	
12	0.455	0.984	1.004	
13	0.409	0.964	0.980	
14	0.389	0.943	0.962	
15	0.376	0.921	0.939	
16	0.336	0.900	0.918	
17	0.287	0.877	0.897	
18	0.280	0.855	0.876	
19	0.234	0.831	0.853	
20	0.224	0.803	0.826	
21	0.181	0.768	0.798	
22	0.153	0.719	0.761	

*Polychoric correlation matrices: 500. Promax rotation, Kappa value of 4. Number of components: 3*.

* Loss of control;

** Financial problems;

*** *Tolerance and withdrawal symptoms*.

**Table 6:** PCA based on polychoric correlations.

**Variable**	**Factor 1**	**Factor 2**	**Factor 3**
1	0.617		
2	0.757		
3		0.780	
4	0.946		
5	0.864		
6	0.565		
7		0.833	
8	0.486		
9	0.591		
10		0.848	
11	0.356		0.454
12			0.364
13			0.879
14			0.624
15			0.821
16	0.554		0.330
17			0.524
18	0.429		0.450
19		0.760	
20			0.879
21			0.565
22			0.583

*Rotated loading matrix (loadings lower than absolute 0.300 omitted)*.

*Polychoric correlation matrices: 500. Promax rotation, Kappa value of 4. Number of components: 3*.

**Table 7:** PCA based on polychoric correlations.

**Variable**	**Factor 1**	**Factor 2**	**Factor 3**
1	0.689	0.453	0.421
2	0.693	0.441	0.261
3	0.461	0.787	0.223
4	0.857	0.371	0.419
5	0.835	0.383	0.470
6	0.683	0.459	0.472
7	0.358	0.818	0.368
8	0.564	0.236	0.454
9	0.694	0.307	0.558
10	0.494	0.875	0.353
11	0.655	0.436	0.691
12	0.581	0.530	0.615
13	0.406	0.304	0.812
14	0.408	0.491	0.686
15	0.477	0.321	0.814
16	0.697	0.308	0.611
17	0.600	0.516	0.723
18	0.593	0.197	0.615
19	0.281	0.754	0.450
20	0.285	0.215	0.735
21	0.512	0.257	0.658
22	0.560	0.439	0.726

*Structure matrix*.

*Polychoric correlation matrices: 500. Promax rotation, Kappa value of 4. Number of components: 3*.

Reliability analysis of the 22-item TMD questionnaire showed acceptable internal consistency, with a Cronbach's alpha of 0.905. Parallel analysis based on principal components analysis extracted three factors that together accounted for 57.96% of the variance. Factor 1 accounted for 41.91% of the variance and was named “Loss of control,” including items 1, 2, 4, 5, 6, 8, 9, 11, 16, and 18. Factor 2 accounted for 8.83% of the variance and was named “Financial problems,” including items 3, 7, 10 and 19. Factor 3 accounted for 7.22% of the variance and was named “Tolerance and withdrawal symptoms,” including items 11, 12, 13, 14, 15, 16, 17, 18, 20, 21, and 22. Cross-loadings were found for item 11 (“When I haven't used my mobile phone for a while, I feel the need to call someone, send an sms or to contact someone through social media”), 16 (“When my mobile phone is in my hand, I can't stop using it”), and 18 (“The first thing I do when I get up in the morning is to see if someone has called me, sent me an SMS or written to me through social media”), and assigned to the single factor with the highest loading. As it was assumed that this was the latent nature of these items, the items were retained in the test.

### Mobile phone use

The mean test total score in the present study was 27.72 for the whole group, with a statistically significant difference in mean score between men (22.48) and women (30.96, *p* < 0.01). One-Way ANOVA also showed a statistically significant difference in mean test total score between the different age groups, with the 15–18 years group having the highest mean test score of 31.03, and the 55 years and older group having the lowest mean test score of 19.40 (*p* < 0.01, η^2^ = 0.068). There was a statistically significant difference in mean test total score between the group rating their subjective dependence at 80% or higher (40.68, *p* < 0.01) and the group rating their subjective dependence at lower than 80% (mean = 25.04). There was also a statistically significant correlation (*p* < 0.01) of 0.612 between test total score and rated subjective dependence, expressed as the absolute percentage reported (0–100), meaning that a higher rated subjective dependence also yielded a higher total test score.

In total, 47 people (3.1%) had a total test score of 58 or higher, and 16 people (1.1%) had a total test score of 66 or higher. Logistic regression revealed a statistically significant (*p* < 0.05) impact of both sex (58 or higher; df 1, OR 3.90 [1.72–8.80]. 66 or higher; df 1, OR 5.06 [1.14–22.50]) and age (58 or higher; df 1, OR 0.59 [0.47–0.73], 66 or higher; df 1, OR 0.21 [0.07–0.62]) in both of these two groups. Being female and at young age increased the odds of scoring higher than the cut-off scores.

### Gambling

Eighty-four (5.5%), 29 (1.9%), and nine subjects (0.6%) endorsed one, two or three gambling items, respectively, such that a total of 122 subjects (8.1%) were categorized as problem gamblers. Furthermore, 11 subjects (0.7%) answered “yes” to the question “Have you ever received professional care, treatment or counseling for gambling problems or gambling disorder?”

Problem gambling was statistically significant associated with a younger age group (chi-square linear-by-linear *p* < 0.001) and with male sex (16.2 and 3.0% problem gamblers among men and women, respectively, *p* < 0.001), and when controlling these variables for one another, both age (df 1, OR 0.83 [0.75–0.93]) and male sex (df 1, OR 6.32 [4.08–9.78]) demonstrated statistically significant associations with problem gambling. When controlling for age and sex, problem gambling was statistically significant associated with the number of daily mobile phone calls (df 1, OR 1.33 [1.07–1.65]), the frequency of using the mobile phone for games (df 1, OR 1.57 [1.37–1.80]), with subjective dependence on one's mobile phone (df 1, OR 1.09 [1.01–1.18]), and the association of problem gambling with the number of friends to communicate with during 1 day (df 1, OR 1.37 [0.99–1.99]) was nearly statistically significant. In contrast, no association was seen between problem gambling and the number of daily text messages, keeping the mobile phone on at night, mobile phone use in bed, and daily hours on social media, when controlling for age and sex. Problem gambling was statistically significant associated with readiness to respond while with friends (df 1, OR 1.56 [1.37–1.90]) or in bed (df 1, OR 1.23 [1.06–1.42]), while it was unrelated to readiness to respond while at work, during lunch/dinner, or when relaxing at home, again controlling for age and sex.

No statistically significant difference was found in relation to mean test total score for the subjects with a history of receiving professional treatment for gambling (42.55 vs. 27.61, NS) and alcohol or other drug use disorder (30.18 vs. 27.63, NS). The total TMD score did not differ between problem gamblers and non-problem gamblers (28.25 vs. 27.67, NS, df 1, OR 1.00 [0.99–1.02]), and in linear regression, the total test score was unrelated to the number of endorsed gambling items (*r* = 0.03, *p* = 0.32). However, the adjusted logistic regression analysis, controlling for age and sex, demonstrated a statistically significant association of TMD score with problem gambling (df 1, OR 1.02 [1.01–1.03], *p* = 0.005).

## Discussion

The main objective of this study was to validate the TMD in a new setting, and in the general population, not exclusively among young people. The study also intended to investigate potential relations between high mobile phone use and problem gambling, or with having a history of having received professional treatment for gambling problems. One main finding was the high internal consistency of the TMD and its correlation with subjective, self-reported level of feeling dependent on one's mobile phone. A second main finding is that problem gambling, which was significantly associated to younger age and male sex, was statistically significant and positively associated with the TMD score, but only after adjusting for age and sex.

The internal consistency of the TMD was acceptable (Cronbach's alpha = 0.905), which may indicate that the items in the questionnaire are measuring the same construct, such as mobile phone dependence. The Cronbach's alpha figure in the present study is similar to the one Chóliz found in his study, with a Cronbach's alpha of 0.94 (Chóliz, 2012). The three factors extracted were named “Loss of control,” “Financial problems,” and “Tolerance and withdrawal symptoms” based on the average content of the items loading to each factor, additionally in an intent to resemble the names of the three factors found in the study by Chóliz (2012). In the same study, the researchers found the three factors together explaining 58.71% of the total variance, in comparison to the figure found in the present study, with the three factors accounting for 57.96% of the variance. This is above a suggested cut-off proportion of 50% of the total variance explained by the retained factors (Streiner, 1994).

In the present study, the two proposed cut-off scores for TMD revealed a prevalence of mobile phone dependence of 3.1 and 1.1% respectively. These figures might be comparable to the prevalence figures found in some previous studies (Götestam and Johansson, 2003; Petry et al., 2005; Toce-Gerstein et al., 2009; McBride et al., 2010). However, prevalence studies of the concept of mobile phone dependence have shown rather big variations, ranging from just a few percent up to more than 20 % (McBride et al., 2010; Long et al., 2016; De-Sola et al., 2017a; Lopez-Fernandez et al., 2017). This could partly be explained by different questionnaires being used, but also different populations and subgroups studied, usually with higher prevalence in children and adolescents.

A statistically significant difference in mobile phone use in relation to sex was found, with women scoring higher than men. If future research provides a clinical picture of mobile phone dependence to be a clinical diagnosis, it might differ from other substance-use- and addictive disorders, as they so far have been found more commonly in men (Ladd and Petry, 2002; Desai et al., 2010; Abbott et al., 2014; Fröberg et al., 2015; Grant et al., 2015, 2016; Rehbein et al., 2015). In contrast to established addictive disorders, an over-use of mobile phones previously has been reported to be more common in girls than in boys, in adolescent populations (Kim et al., 2015; Randler et al., 2016; Lopez-Fernandez et al., 2017). This is consistent with the sex difference seen in the present study.

The concept of an addictive disorder related to excessive mobile phone use is however far from controversial. Billieux and colleagues questions the framework of mobile phone dependence studies, accusing these for assuming a priori that a mobile phone dependence syndrome exists, and that tests were created in order to verify its existence. Furthermore, the same authors question to what extent already established substance use disorder criteria can be directly translated and used within a potential mobile phone dependence context (Billieux et al., 2015b). The use of cross-sectional studies to validate mobile phone dependence has also been questioned. A call for longitudinal studies, together with neurobiological evidence of tolerance in individuals proposed to suffer from it, have been proposed as an alternative research approach (Billieux et al., 2015a). However beneficial this approach would be, the research of mobile phone use and its possible pathological usage patterns is a somewhat new phenomenon, which makes prevalence and cross-sectional studies a crucial part in gaining a basic understanding of its possible components. Several studies have highlighted different potential constituents of problematic mobile phone use, such as excessive social media use, e-mailing and Internet access, gaming, worsened social functioning, a fear of missing out, and texting in dangerous situations, Roberts et al. (2014), Chen et al. (2017), Lopez-Fernandez et al. (2017), Kuss et al. (2018), and Upreti and Musalay (2018). The TMD demonstrated acceptable psychometric characteristics in this study. In addition, it also indicated a potential link to the more clearly established problem construct describing an excessive or problematic use of gambling for money.

Moreover, it is essential to consider the possibility of cohort effects and that some behaviors seem to be transient and sometimes episodic rather than chronic, as Thege and co-workers showed in their longitudinal study (Thege et al., 2015). Interestingly, a Spanish study found that university students reported an increase in problematic mobile phone use and associated negative consequences in 2013 compared to their 2006 counterparts (Carbonell et al., 2018). In the present study, 3.0% of the subjects rated their subjective mobile phone dependence at 100%. However, this is clearly not equal to 3.0% of the subjects suffering from a psychiatric disorder. As Stein et al. (2010) point out, as there are no objective biomarkers to define most of the psychiatric disorders today, it is essential to look at whether the behavior studied is causing a clinically significant impairment or not (Stein et al., 2010).

Another main finding was the association between total TMD score and problem gambler status, although this association appeared only when controlling for sex and age, as the actual values in TMD score were virtually the same across gambling groups. An individual scoring one out of three on the NODS-CLiP questions was considered a “problem gambler,” yielding a lifetime 8-% prevalence of problem gambling. Although measured with a different instrument, previous data have reported a lifetime prevalence of problem gambling of around 2.5% in Sweden, but with markedly higher number in younger age groups (Abbott et al., 2014). The age distribution in the present study was skewed toward younger aged groups, which probably accounts for part of the heightened prevalence of problem gambling found. However, again, this study did not intend to reach a nationally representative sample of adults, and it cannot be excluded that a self-test for possible mobile phone dependence might have attracted problem gamblers to a somewhat larger extent than non-problem gamblers. It is worth mentioning that although some of the odds ratios reported in the study may appear low, many of the variables analyzed are divided in scale steps, as why the added effect might be stronger.

The relationship between lifetime problem gambling and mobile phone behavior was markedly different when not adjusting for age and sex, such that problem gamblers and non-problem gamblers reached virtually the same total score on the TMD. In contrast, while female sex was associated with TMD score and male sex was associated with problem gambling, and while younger age predicted both TMD and problem gambling, the adjusted analysis revealed a statistical association between TMD score and increased likelihood of reporting a lifetime gambling problem. In addition to these overall measures, several other items describing mobile phone behavior (apart from the TMD) separated problem gamblers from non-problem gamblers, although this picture is not unanimous and harder to interpret. For example, lifetime problem gamblers reported more mobile phone calls per day and reported communicating with more people daily, whereas they did not report sending more text messages per day. The association with the frequency of using games in the mobile phone (and where games for money were included in the question, along with different types of videogames), may not seem surprising. For other associations seen here, more research will be needed in order to establish how different aspects of mobile phone use may differ between problem gamblers and the rest of the population. To the best of our knowledge, the present study is the first one to evaluate the potential association between a structured measure of mobile phone dependence, or an addiction-like mobile phone behavior, and problem gambling. Clearly, more research is needed in this area, in order to replicate the present findings of separate items being related to gambling behavior, as it cannot be excluded that certain components of a pattern of mobile phone use may be more associated with gambling behavior. Likewise, the direction and causality of such an association cannot be established from the present study, where problem gambling may have occurred at any time during a person's life, whereas the mobile phone pattern referred to the current situation. Clearly, gambling for money has been available for several decades more than has the use of mobile phone devices, and longitudinal studies, addressing gambling behavior and mobile phone behavior simultaneously, are needed. However, the findings of the present study do indicate a potential link between a more intense or exaggerated mobile phone use and the most clearly established non-substance addictive behavior, gambling, lending some support to potentially shared characteristics between problem gamblers and individuals with a more intense mobile phone use.

### Strengths and limitations

A strength of the present study is that subjects were able to fill in the questionnaire online, through their smartphone, tablet or computer. This allowed a large number of people to answer the questionnaire anonymously. As with all questionnaires, there is the possibility of bias. In the case of the TMD and the other questions, it is plausible to assume that social desirability bias as well as cultural normativity might have influenced some of the answers, as addiction have since long held a social stigma. The sampling method used in the study did not allow us to control the correct and honest completion of the questionnaire. However, use of Mahalanobis distance, the elimination of incompletely filled out questionnaires (*n* = 254) together with the large number of subjects, ought to have corrected somewhat for this. Earlier studies conducted on potential mobile phone dependence have only targeted children or adolescents. To the best of our knowledge, mobile phone use behavior has not yet been studied across different age groups. This study included a wide range of age categories, making it possible to make comparisons on patterns of use.

A limitation of the present study is that the majority of the subjects filling out the questionnaire were women (61.7%). One explanation to this might be the common finding that women in general seem to show a higher response rates in surveys (Smith, 2008; Aerny-Perreten et al., 2015; Glass et al., 2015). Future studies should take this into consideration, as this majority of female respondents in the present study provide a non-weighted sample. Another limitation of the study is that the motives and feelings behind usage patterns of the subjects were not revealed. It might be that some individuals use the mobile phone as a way of coping with negative or other stressful emotions, as seen in a study of IGD, where the “healthy users” mostly played for social reasons, while the “risk players” more commonly played as a way of escape and/or as a coping mechanism (Kim et al., 2016).

In conclusion, the results of this study show that the TMD has acceptable internal consistency and that the test correlates with subjective dependence. The test may be useful in future screening for mobile phone use behavior, but a confirmatory factor analysis to further evaluate the instrument is recommended. However, more research is needed to further investigate the individual reasons for using the mobile phone in ways that may be considered an addiction. Moreover, in the present study it was noted that many people experience a subjective dependence, and that they assume they would experience withdrawal symptoms if unable to use their mobile phone. Finally, problem gambling was shown to be associated with mobile phone behavior, including the total TMD score, and although this link needs to be interpreted with great caution, it cannot be excluded that individuals with problematic gambling and with an addiction-like mobile phone behavior may share similar characteristics. More research is needed in order to examine the role of excessive mobile phone behavior in society, and its potential associations with other behavioral addictive disorders.

## Ethics statement

All procedures performed in studies involving human participants were in accordance with the ethical standards of the institutional and/or national research committee and with the 1964 Helsinki declaration and its later amendments or comparable ethical standards. Informed consent was obtained by all individual participants included in the study. As none of the study participants were younger than 15 years old, parental approval was not needed. The project was approved by the regional ethics committee Lund, Sweden (file number 2016/53).

## Author contributions

AF: Corresponding author, main author; MC: Founder of the TMD. Provision of feedback on the statistics. AH: Academic supervisor, providing feedback on the manuscript.

### Conflict of interest statement

The authors declare that the research was conducted in the absence of any commercial or financial relationships that could be construed as a potential conflict of interest.
